# Differential Gene Expression during Larval Metamorphic Development in the Pearl Oyster,* Pinctada fucata*, Based on Transcriptome Analysis

**DOI:** 10.1155/2016/2895303

**Published:** 2016-10-23

**Authors:** Haimei Li, Bo Zhang, Guiju Huang, Baosuo Liu, Sigang Fan, Dongling Zhang, Dahui Yu

**Affiliations:** ^1^Key Laboratory of South China Sea Fishery Resources Exploitation & Utilization of Ministry of Agriculture, South China Sea Fisheries Research Institute, Chinese Academy of Fishery Sciences, Guangzhou, Guangdong 510300, China; ^2^Shanghai Ocean University, Shanghai 201306, China; ^3^Jimei University, Xiamen, Fujian 361021, China

## Abstract

*P. fucata* experiences a series of transformations in appearance, from swimming larvae to sessile juveniles, during which significant changes in gene expression likely occur. Thus,* P. fucata* could be an ideal model in which to study the molecular mechanisms of larval metamorphosis during development in invertebrates. To study the molecular driving force behind metamorphic development in larvae of* P. fucata*, transcriptomes of five larval stages (trochophore, D-shape, umbonal, eyespots, and spats) were sequenced using an Illumina HiSeq™ 2000 system and assembled and characterized with the transcripts of six tissues. As a result, a total of 174,126 unique transcripts were assembled and 60,999 were annotated. The number of unigenes varied among the five larval stages. Expression profiles were distinctly different between trochophore, D-shape, umbonal, eyespots, and spats larvae. As a result, 29 expression trends were sorted, of which eight were significant. Among others, 80 development-related, differentially expressed unigenes (DEGs) were identified, of which the majority were homeobox-containing genes. Most DEGs occurred among trochophore, D-shaped, and UES (umbonal, eyespots, and spats) larvae as verified by qPCR. Principal component analysis (PCA) also revealed significant differences in expression among trochophore, D-shaped, and UES larvae with ten transcripts identified but no matching annotations.

## 1. Introduction

Metamorphosis is a series of key steps in the process of larval development, the success of which affect the survival of the organism. Metamorphosis is prevalent in insects, amphibians, some fishes, and many marine invertebrates, such as barnacles, sponges, shellfishes, shrimps, and echinoderms. Similar to most benthic marine invertebrates, the pearl oyster (*Pinctada fucata*) has a microscopic, free-swimming larval phase in their complex life cycle [1]. Oyster larvae spend several weeks in the water column before attaining competency to attach and metamorphose, commencing their sessile life. The developmental processes of* P. fucata*, from swimming larvae to sessile spats, have been classified into six stages: fertilized egg, trochophore, D-shaped, umbonal, juvenile, and adult stages [2]. In oysters, the transition from free-swimming larvae to the attached juvenile form often requires morphological, physiological, structural, and functional changes, which are under genetic regulatory control [3]. Therefore, the identification of key developmental genes involved in the metamorphosis of* P. fucata *larvae, as well as characterizing their expression patterns, is important to understand the molecular mechanism of metamorphic development of this economically important species.

Numerous studies have been conducted to explore the mechanisms of hormones, neurotransmitters, genes, and signaling pathways that regulate larval metamorphic development. Some studies have demonstrated that eight superfamily genes showed differential expression during the metamorphosis of* Ciona intestinalis* [4–7]. Several homeobox-containing genes were found to be responsible for larval metamorphic development in* Haliotis rufescens* [8–10]. In addition, abnormal dopamine and adrenaline were observed in the larval attaching stage of the Pacific oyster,* Crassostrea gigas* [11], while a different study observed increased expression of a molluscan growth and differentiation factor (mGDF) in the metamorphosing stage of the same organism [12]. These findings indicate the diversity of genes involved in the transitions of larval forms.

However, previous studies have focused on changes in a small number of genes and have provided a fragmented view of the genetic modulation of larval metamorphosis. Recent developments in sequencing technology have allowed for the development of new genomic tools, which can provide a more global view of changes in gene expression over the course of larval developmental stages [13–16]. In terms of genome-wide studies, transcriptome analyses are considered to be an ideal choice for obtaining comprehensive information regarding animal development and growth [17, 18]. For* P. fucata*, the draft genome [19] and tissue transcriptomes [20–23] have been recently reported. Based on the transcriptomic sequences from a mixture of nine developmental stages of* P. fucata* [19], biomineralization-related gene expression profiles during larval development have been investigated [24, 25] and genes involved in body patterning [26], transcription factors [27], and homeobox genes [28] have been identified. Nonetheless, developmentally important genes and their expression patterns during the larval stages of developing* P. fucata *have not been systematically studied at the transcript level to date. In the present study, the transcriptomes of five larval stages (trochophore, D-shape, umbonal, eyespots, and spats) and six tissues (gill, adductor muscle, hepatopancreas, mantle, hemocytes, and pearl sac) from* P. fucata* were sequenced using Illumina HiSeq 2000, with an emphasis on the molecular mechanisms underlying larval metamorphic development. This study aims to provide a valuable insight into the mechanisms of genetic modulation over the course of larval metamorphic development for* P. fucata* as well as for other molluscan species.

## 2. Materials and Methods

### 2.1. Larval Culture and Sample Collection

Larvae of* P. fucata *were bred (using several females and males of a selectively bred F3 generation as parents) through artificial insemination on March 10, 2013, in Sanya, Hainan Island, China, as described by Fujimura et al. [2]. Fertilized eggs were incubated in a 1000 L tank at 24°C. After removing nondeveloping embryos and dead larvae, trochophore, D-shaped, umbonal, eyespots, and spats larvae stages were harvested with filtering net at 12 h, 36 h, 11.5 d, 18.5 d, and 23.5 d after fertilization, respectively, and immediately preserved in RNA later (TaKaRa Bio Inc) until RNA extraction. Meanwhile, RNAs of six tissues (gill, adductor muscle, hepatopancreas, mantle, hemocytes, and pearl sac) of three other adult animals were sequenced for a more robust assembly.

### 2.2. RNA Extraction and cDNA Library Preparation

Following the manufacturer's instructions, total RNA was extracted from five developmental stages (each stage with thousands of larvae) and six tissues using Trizol and RNAs of each type of tissues of the three individuals were mixed by equal weight. RNA integrity and quantity were confirmed by lab-on-chip analysis using a 2100 Bioanalyzer (Agilent Technologies, Santa Clara, CA, USA) and visualized on a 1% agarose gel. Then cDNA was synthesized using the mRNA fragments as templates as usual and was sequenced by the BGI (Shenzhen, China) using the Illumina HiSeq 2000 system (San Diego, CA, USA) (PE100).

### 2.3. Sequence Assembly and Annotation

After filtering out low quality sequences (containing more than 5% ambiguous “N” nucleotides or >20%  *Q*≦10 reads) and the removal of adapters from raw data, clean sequence data was assembled into unigenes using Trinity software and subsequently clustered by TGICL v2.1 (-l 40 -c 10 -v 20) [29]. Phrap (-repeat_stringency 0.95 -minmatch 35 -minscore 35) (Release 23.0) was used to produce the longest sequence possible (http://www.phrap.org/). Assembled unigenes were annotated based on the Nr, Swissprot, KEGG, and COG databases. The sequence direction and amino sequence of the predicted coding region (CDS) of unannotated unigenes were determined using ESTScan with default settings [30]. Functional annotations and classifications were performed by using Blast2GO [31] and WEGO [32] (*E* value threshold 1 × 10^−5^), respectively.

### 2.4. Normalization and Quantification of Gene Expression

Sequencing reads were mapped to the assembled reference sequence using SOAP aligner/soap2 (-m 0 -x 500 -s 40 -l 35 -v 5 -r 2) [33], a tool designed specifically to assemble short sequence alignments. The coverage of reads from a given gene was used to calculate the expression level of that gene, which was measured by fragments per kilobase exon per million fragments (FPKM) [34], with the following formula:

(1) $$\begin{array}{c} FPKM=\frac{10^{6}C}{NL/10^{3}}, \end{array}$$

where FPKM is the expression level of a unigene, *C* is the number of fragments that uniquely aligned to the unigene, *N* is the total number of fragments that uniquely aligned to all unigenes, and *L* is the number of bases in the CDS of the unigene. The FPKM method eliminates the influence of sequences of differing lengths and coverage level on the calculation of gene expression. Therefore, the calculated gene expression can be directly used for comparing the difference in gene expression between samples.

### 2.5. Differential Gene Expression (DEGs) across Developmental Stages

Differential gene expression among different larval stages was carried out via principal component analysis (PCA) using the R package (http://www.r-project.org/) according to the manual. The pairwise differential expression conducted by edgeR, with a threshold of the false discovery rate (FDR) ≤ 0.001 and an absolute value of log 2 Ratio ≥ 1, was used to judge the significance of differences in gene expression. Trends in the expression of all differentially expressed genes were sorted using STEM (Short Time-Series Expression Miner, v1.3.8) [35]. Functional annotation and classification of genes involved in significant trends were performed by using Blast2GO [31] and WEGO [32], respectively. The enriched metabolic pathways or signal transduction pathways of genes were identified based on the KEGG database [36].

### 2.6. Identification and Expression Profile of Genes Involved in the Larval Metamorphic Development of* P. fucata*

According to annotations by Nr and Swissprot, development-related genes were identified with those that had been previously identified as keywords in the significant trends from the prior step. If several unigenes were assigned to the same reference gene, the sequence with the lowest *E* value (Nr and Swissprot annotation *E* value) was selected as a representative. Then, the heatmap 2 module of the gplots package in R (https://cran.r-project.org/web/packages/gplots/index.html) was used to perform the clustering analysis of gene expression on the normalized, filtered sequences to identify genes that were significantly different among the five developmental stages.

### 2.7. qPCR Verification of Expression Trends of Development-Related Genes

In order to verify the integrity of the transcriptome sequences and the expression levels as revealed by RNA-Seq, eight development-related genes were selected randomly for qPCR verification. The genes and respective primers are given in Table 1. qPCR was performed using an Eppendorf real-time- (RT-) PCR system (Eppendorf, Hamburg, Germany) using a SYBR(R) Premix Ex TaqTM kit (TaKaRa) according to the manufacturer's protocol. Transcript levels of target genes were normalized against the level of a reference gene (18S rRNA). The qRT-PCR reactions were performed under the following conditions: 94°C for 5 min (one cycle), 94°C for 20 s, 50°C to 60°C for 20 s, and 72°C for 20 s (50 cycles). The comparative CT method (2^−ΔΔCT^ method) was used to determine the relative mRNA abundance [37].

## 3. Results

### 3.1. Sequence Assembly and Annotation

**Figure 1:**
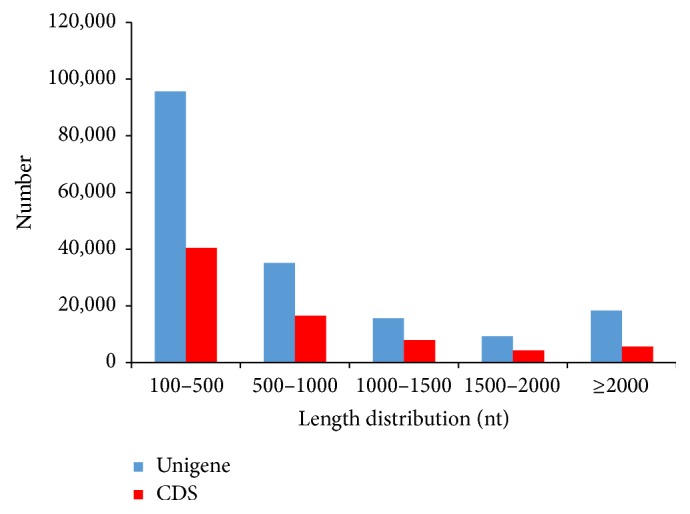
Length distribution of unigenes and CDS.

Over 55 million reads per sample were generated with a base call accuracy (Q20) of over 97%. The number of contigs varied from 118,010 to 215,808, with a median length (N50) of 352 to 582 bp (Table 2). The number of unigenes varied from 51,102 to 113,516 among samples, with the mean length ranging from 536 to 689 bp, while N50 ranged from 495 to 1,025, respectively. In total, 174,126 unigenes were assembled with a mean length of 866 bp and an N50 of 1, 569 (based on 11 samples). Most unigenes were 100–500 bp long, and 26% were greater than 1,000 bp (Figure 1).

**Figure 2:**
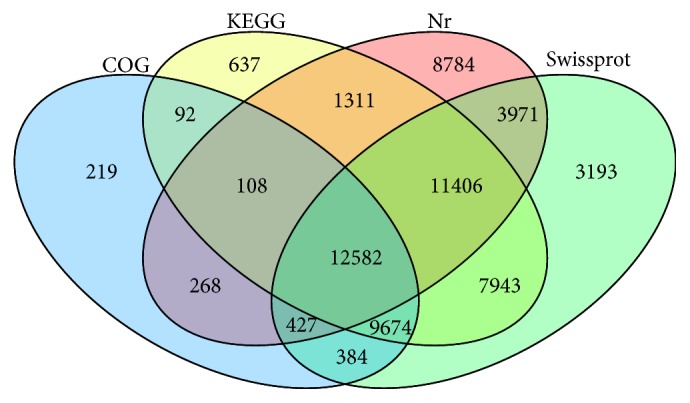
Number of genes annotated by COG, KEGG, Nr, and Swissprot terms based on five larval stages and six tissues of* Pinctada fucata*.

In total, 60,999 unigenes were annotated (Table 3) and 74,912 CDSs (43.02%) were predicted (13,966 predicted by ESTScan) (Figure 1, Table 3). Different databases annotated different numbers of unigenes (Table 3), where the most unigenes (49,580) were annotated by Swissprot database and the least (13,465) by the GO database (Table 3). Numbers of specific and shared unigenes annotated by COG, KEGG, Nr, and Swissprot terms can be visualized in Figure 2. Among them, 12,582 unigenes were annotated by the four databases and 8,784 were annotated specifically by Nr (Figure 2). Both KEGG and Swissprot analyses shared the most unigenes (41,605) and COG and Nr shared the least (13,385).

**Figure 3:**
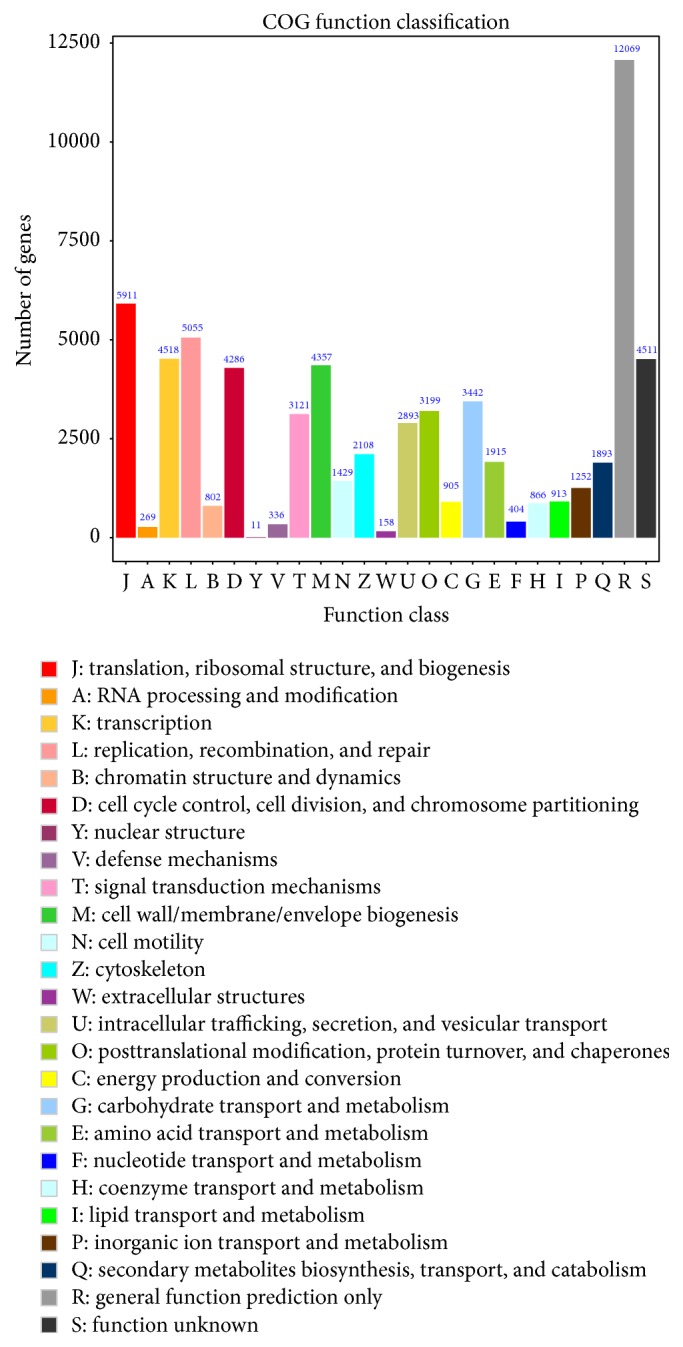
COG function classification of all unigenes.

**Figure 4:**
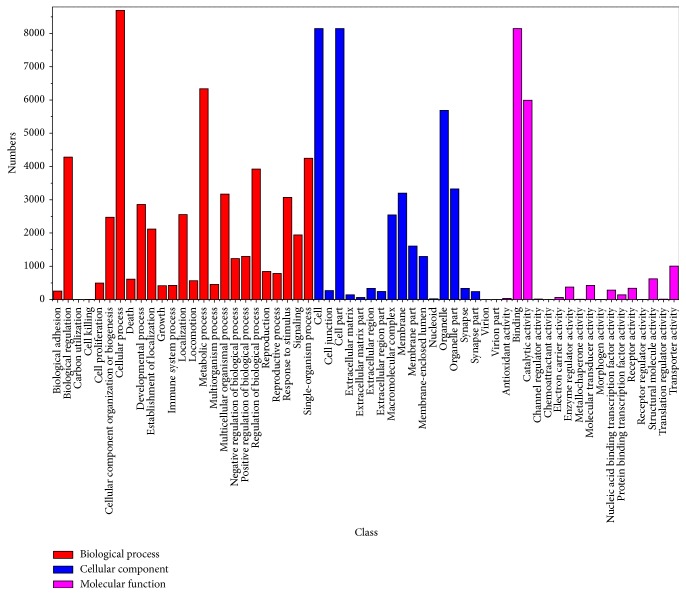
GO categories of unigenes. 13,465 of 174,126 unigenes were assigned to GO annotation and divided into three categories: biological processes, cellular components, and molecular functions.

The 23,754 COG-annotated unigenes can be further classified into 25 functional groups, half of which were sorted into the “general function” group (Figure 3). The GO analysis revealed that 10,165 unigenes were attributed to biological process, 8,442 unigenes to cell components, and 10,588 unigenes to molecular function (Figure 4). The top 26 KEGG pathways are summarized in Table 4. Most unigenes (5,184 out of 43,753) were involved in metabolic pathways and 1,401 unigenes were involved in calcium signaling pathways, some of which may be involved in shell formation. Finally, many unigenes in the top 26 KEGG pathways were involved in immune pathways.

### 3.2. Differential Gene Expression (DEGs) and Expression Trends during Developmental Stages

**Figure 5:**
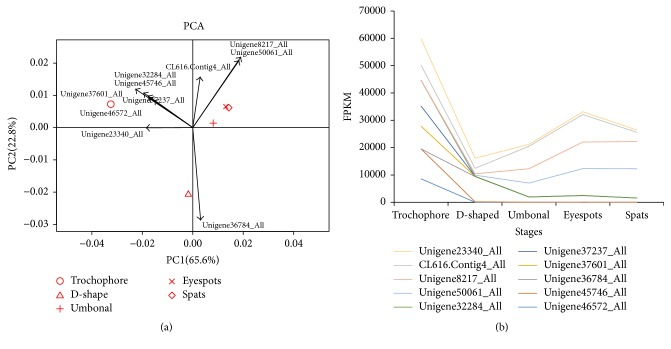
The distinction between five developmental stages as indicated by (a) principal component analysis and (b) expression levels of 10 representative genes, identified to be responsible for the distinction among the developmental stages.

Principal component analysis (PCA) revealed that differences in the expression of unigenes were vast among trochophore, D-shaped, and UES (umbonal, eyespots, and spats) larvae, but small within UES stages (Figure 5(a)). Based on gene effects, measured by the first principal component value, a total of 10 transcripts with unknown functions were identified to be key factors involved in the larval development of* P. fucata*. Differences in the gene expression of these transcripts were the greatest in trochophore, D-shaped, and UES larvae. They were relatively highly expressed in trochophore larvae and then downregulated during the D-shaped stage, and some were subsequently upregulated during the UES stage, including Unigens23340_All, Unigene8217_All, Unigene50061_All, and Cl616_All (Figure 5(b)).

**Figure 6:**
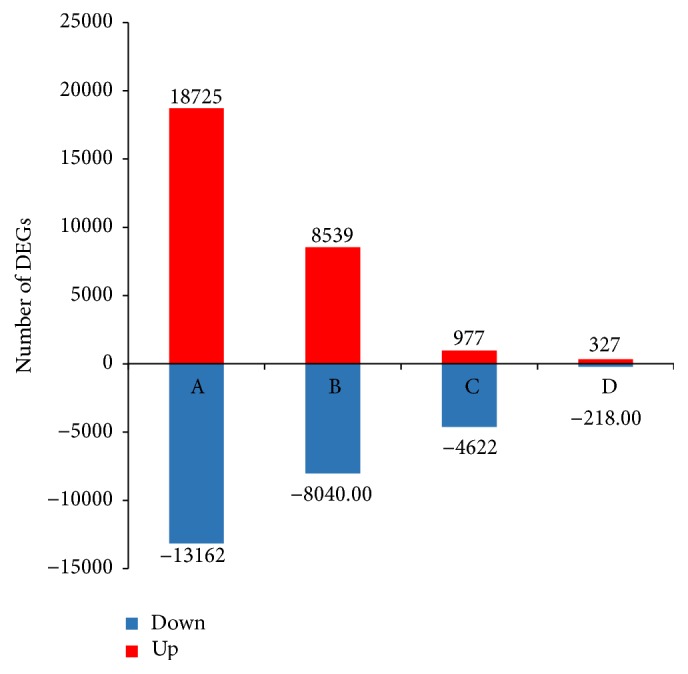
Numbers of up- (red) and downregulated (blue) unigenes. The numbers on column indicate the quantity of up- (red) and downregulated (blue) genes. The results of four comparisons are shown. The signal intensities of each feature of the DEGs are plotted on a logarithmic scale. Statistical criteria for designation of genes as up- or downregulated are outlined in the methods.

**Figure 7:**
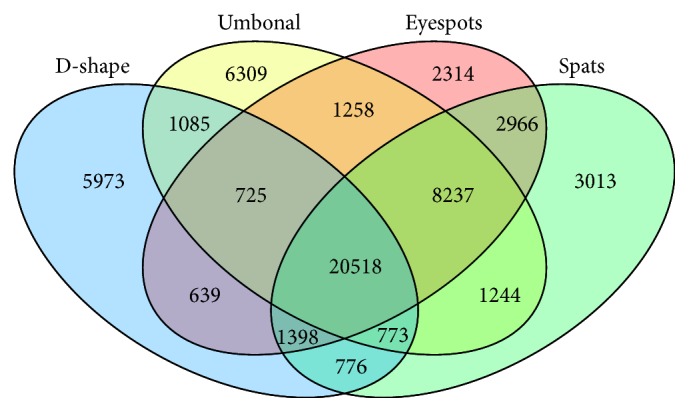
Specific and shared genes in five developmental stages of the pearl oyster,* Pinctada fucata*.

**Figure 8:**
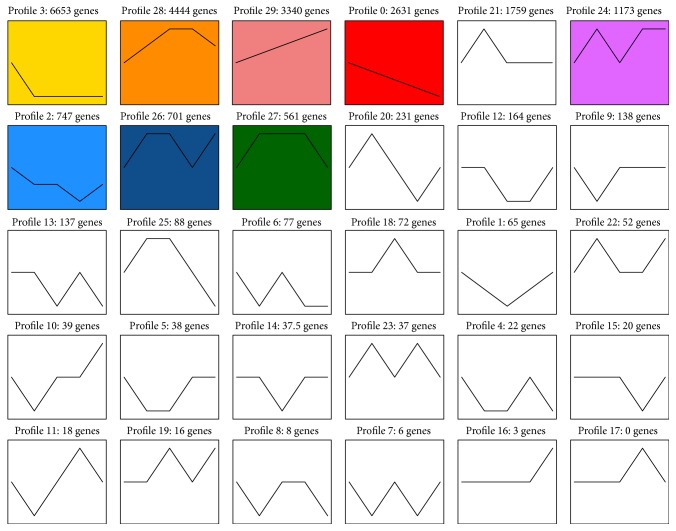
Expression trends of unigenes across trochophore, D-shaped, umbonal, eyespots, and spats larval stages of* Pinctada fucata*. The profiles were ordered based on the *P* value of the number (at bottom-left corner) of genes assigned versus expected. Color square frames denote significant profiles (*P* ≤ 0.01). Each graph displays the mean pattern of expression (black lines) of the profiled genes. The number of profiles in each cluster is indicated in the top left corner. The *x*-axis represents stages and the *y*-axis represents log 2-fold change of gene expression.

The numbers of up- and downregulated unigenes were also much greater during early stage transitions (Figure 6), consistent with the results of the PCA. From trochophore to D-shape larvae, there were 18,725 unigenes upregulated and 13,162 downregulated. In total, there were 57,228 DEGs among the five developmental stages (Figure 7). Additionally, 17,609 genes were preferentially expressed at a single developmental stage, which indicates that they play an important role in the corresponding developmental stage, while 39,619 were expressed preferentially during more than two stages. A total of 20,518 genes were differentially expressed in all five of the development stages. All differentially expressed genes were sorted into 29 expression trends (Figure 8), of which eight trends were significant, comprising over 45% of the total DEGs. Furthermore, 6,653 unigenes were expressed highly only during the trochophore stage. Across the five stages, 3,340 unigenes were expressed in an increasing pattern, while 2,631 unigenes were expressed in a decreasing pattern.

### 3.3. Functional Enrichment Analysis

A functional enrichment analysis of the unigenes from the eight significant trends showed that there were 104, 54, and 46 GO terms for biological processes, molecular functions, and cellular components, respectively, identified for GO function enrichment (see Supplementary Table 1 in Supplementary Material available online at http://dx.doi.org/10.1155/2016/2895303), and 272 pathways identified for KEGG pathway enrichment (Supplementary Table 2). For GO enrichment data, trends 0, 2, 3, 24, 26, 28, and 29 were involved in biological processes, where most unigenes belonged to trend 3 and were related to various metabolic processes. Trends 0, 3, 24, 28, and 29 were involved in molecular function, where most unigenes fell within trends 0, 3, and 28, and were related to binding and catalytic activity. Only trends 0, 3, 28, and 29 were involved in cellular components, and most unigenes fell within trend 3 and were involved in processes related to membranes and organelles.

Trends 0, 2, 3, 24, 26, 27, 28, and 29 were implicated in KEGG pathway enrichment. For 272 significant enriched pathways, 81 pathways were observed in trend 29, 77 in trend 28, 30 in trend 27, and 27 in trend 26 (Supplementary Table 2). In trends 28 and 29, most unigenes were involved in immune responses.

In trend 0, GO enrichment showed that macromolecule metabolic processes were the dominant groups in biological process, followed by positive regulation of biological process. For pathways involved in molecular function, DNA binding was the most representative category, while for cellular component pathways, all of the genes participate in processes integral to the inner mitochondrial membrane and are intrinsic to the inner mitochondrial membrane. In the KEGG category, spliceosomes were prevalent, followed by genes involved in the cell cycle.

In trend 3, GO enrichment data showed that 6,653 DEG unigenes were further categorized into 43 functional groups; among them, macromolecule metabolic processes were the dominant groups in biological process, followed by cellular macromolecule metabolic processes. In the molecular function category, a high percentage of genes came from the binding and protein binding groups. Spliceosomes were the most representative, followed by RNA transport and regulation of the actin cytoskeleton.

In trends 27 and 28, GO enrichment reveled that there were no significant categories. However, the calcium signaling pathway, hedgehog signaling pathway, and insulin signaling pathway were significantly enriched in the KEGG database, as they are all involved in early development. In trend 28, small molecule metabolic processes were the dominant group in biological process followed by ion transport. Catalytic activity was the most prevalent in the molecular function category, followed by transporter activity and transmembrane transporter activity. In cellular component pathways, membrane was the most representative, followed by plasma membrane. In KEGG enrichment categories, we also found genes related to the calcium signaling pathway in trend 27.

In trend 29, translational elongation was the only enriched category for biological processes, while three categories were enriched in molecular function, including genes involved in oxidoreductase activity, catalytic activity, and lyase activity. In cellular component pathways, five categories were enriched, while vacuole was the dominant group, followed by lytic vacuole and lysosome groups. Genes in trend 29 were enriched in only one KEGG pathway, translational elongation, with a significant *E* value.

### 3.4. Identification and Expression Profiling of Genes Involved in the Larval Metamorphic Development of* P. fucata*

**Figure 9:**
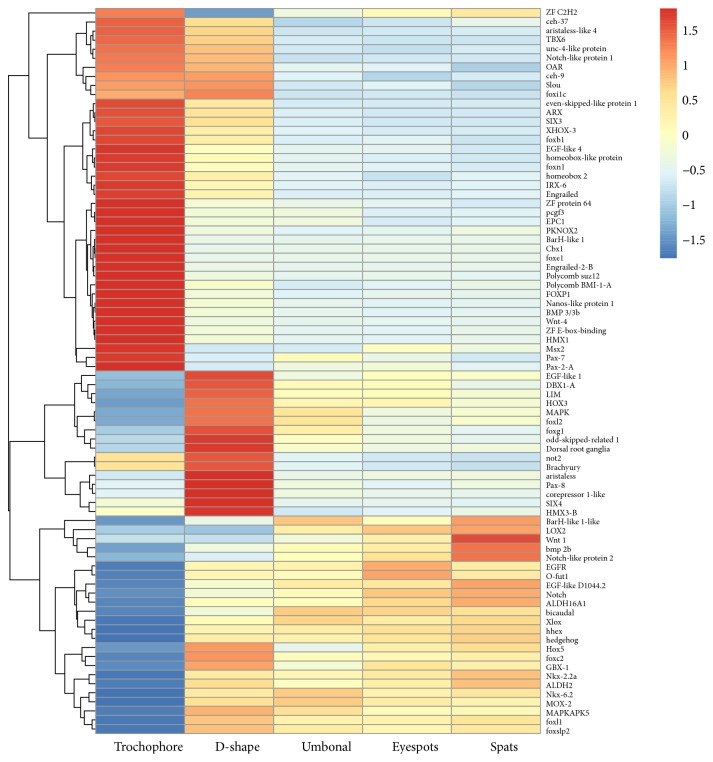
Clusters of expression patterns of development-related genes across five larval stages in* Pinctada fucata*.

**Figure 10:**
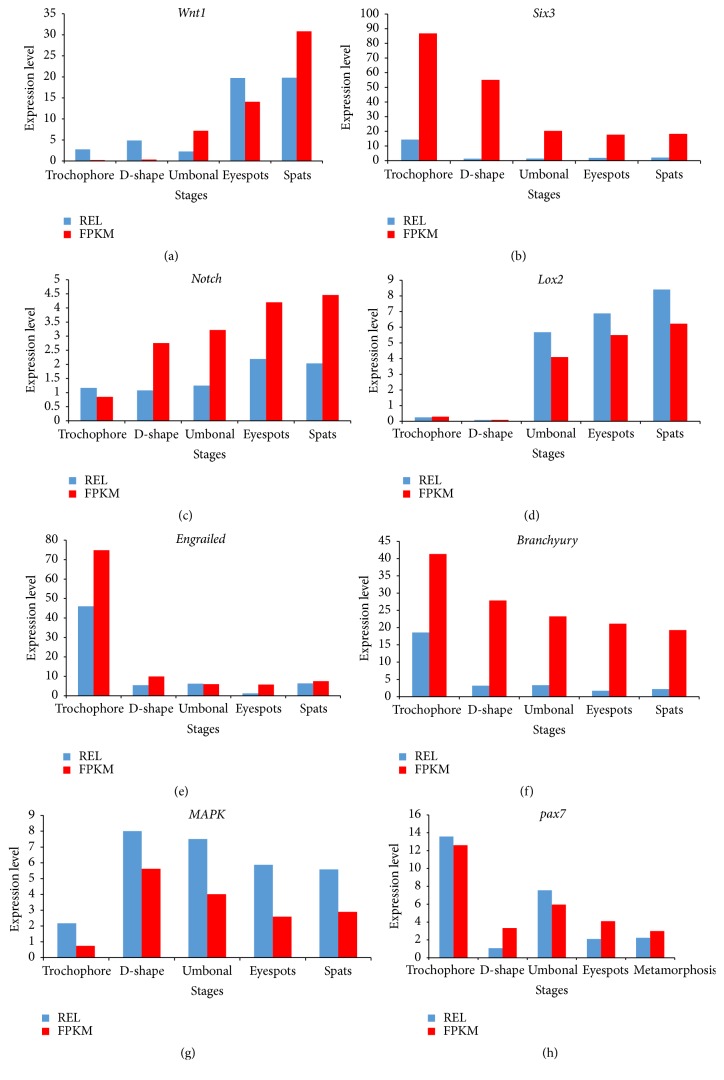
Expression changes in (a)* Wnt1*, (b)* Six3*, (c)* notch*, (d)* lox2*, (e)* engrailed*, (f)* branchyury*, (g)* MAPK*, and (h)* pax7* in trochophore, D-shaped, umbonal, eyespots, and spats larval stages by qPCR.

In total, 80 development-related candidate DEGs were identified and summarized in Table 5, which can be mapped to known developmentally important genes, including several homeobox genes, and can be sorted into 10 trends: trend 0 (25 unigenes), trend 28 (16), trend 3 (15), trend 29 (9), and six other trends (1–5). Cluster analyses suggested that most development-related candidate genes were highly expressed in the early developmental stages (Figure 9), including* engrailed-2-B*,* pax* family,* fox* family members* e1* and* p1*,* Wnt-4,* and* BMP3/3B *upregulated in the trochophore stage and* LIM*,* foxg1*,* Hox3*,* bicaudal*,* hedgehog*,* EGFR*,* foxl2*, and* bmp 2b *genes upregulated from the D-shaped stage until the eyespots stage. In the spats stage,* wnt1* and* notch-like protein 2* gene were upregulated. The qPCR showed that the trends in the expression of selected genes (Figure 10) were consistent with the expression trends indicated by the trend analysis of RNA-Seq data (Figure 9), indicating that the sequence data in our study are reliable.

## 4. Discussion

Not only does the pearl oyster,* P. fucata*, make an ideal model organism for studies of biomineralization, but also it is a good model to study the early stage metamorphic development of invertebrates. In this study, we sequenced the transcriptomes of five developmental stages in* P. fucata*, with the aim of developing a better understanding of the molecular mechanisms driving the change of one larval stage to the next during early life history. In our study, the* de novo* assembly was performed with six tissue transcriptomes, as the draft genome of* P. fucata* is not complete [19]. As a result, we obtained 174,126 unigenes, with a mean length of 866 bp. A total of 60,999 unigenes (35%) were annotated, a value slightly higher than previous reports [21–23, 38]. Poor annotation efficiencies have been widely prevalent in many marine organisms, likely owing limited genomic resources from aquaculture species in public databases to date [21–23, 38]. Alternatively, poor annotation efficiencies could be the result of the short length of the assembled unigene sequences [22] and great divergence among the genomes of marine organism. Similar scenarios have been reported in other marine organisms [39, 40]. In the KEGG annotation, we observed that many pathways were related to immunity, indicating that innate protection is vital in the early developmental stages.

Differential gene expressions (DEGs) occurred mainly during early stage transitions (Figure 6). Most genes were up- or downregulated from trochophore to D-shaped and from D-shaped to umbonal stages, indicating that processes associated with these transitions are very complicated. Principal component analyses yielded consistent results, where we identified 10 unigenes attributed to the divergence among trochophore, D-shaped, and UES (umbonal, eyespots, and spats) stages in* P. fucata*, being highly expressed in the trochophore stage. However, no functional annotations match these functionally important sequences, indicating that further research would help to elucidate the molecular mechanism of metamorphosis in this species in the future.

The analysis of expression trends indicated that 12,009 of 13,277 unigenes are sorted into eight significant expression trend groups. Among the significant trends, there were 10,031 (trends 3, 0, and 2), 11,978 (trends 28, 29, 21, 24, 26, and 27), 9,046 (trend 28, 29, 26, and 27), 9,518 (trend 28, 29, 24, and 27), and 5,214 (trend 29, 24, and 26) unigenes displaying increased expression in trochophore, D-shaped, umbonal, eyespots, and spats stages, respectively. This conveys that more genes are expressed in the early stages, consistent with the DEG and PCA analyses in our study. Particularly, 6,653 unigenes (trend 3) were highly expressed only in the trochophore stage, 3,340 unigenes (trend 29) expressed in an increasing pattern over the course of development, and 2,631 unigenes (trend 0) expressed in a decreasing pattern. These genes are worth further investigation.

The KEGG pathway enrichment analysis indicated that most unigenes in trend 3 were involved in pathways of spliceosome or RNA transport, indicating that, in the early stage of* P. fucata*, RNA synthesis is more predominant. On the contrary, genes in trend 29 showed significant enrichment in translational elongation pathways, suggesting that protein synthesis is more and more prevalent during larval development. In trends 27 and 28, a large number of unigenes were involved in the calcium signaling pathway, synchronizing with the shell formation of prodissoconchs I and II in D-shaped and umbonal stages [24, 41]. In addition, immune pathways were also enriched, indicating that innate protection is important during the entire course of larval development [3, 42, 43].

In our study, 80 known development-related, differentially expressed unigenes were identified throughout the five larval stages (Table 5). Half of them were homeobox-containing genes, including genes known to be involved in the development of body patterning (*engrailed*,* SIX3*,* Pax-7*,* LIM*, and* Hox *family members), suggesting that these genes play important roles in the metamorphic changes of* P. fucata* larvae. Nearly half of the homeobox-containing genes were upregulated in trochophore and D-shaped stages (Figure 9). We identified two* Hox* genes,* Hox5* and* Hox3* (Figure 9), which are highly expressed in D-shaped veliger, indicating that they are involved in the growth of D-shaped larvae. We also found early developmentally relevant signaling molecules such as Hedghog, TGF*β*, and Wnt family, which are known to play important roles in axis formation, muscle differentiation, and nervous system development [26]. Recent evidence has suggested that classic morphogens, such as Wnts, TGF*β*/BMP family members, and Hedgehogs, may all serve as axon guidance cues for a variety of axons in different organisms [44]. Several studies have provided increasing evidence that* Sonic hedgehog *(*Shh*) is an important axon guidance cue throughout vertebrate neural development [45, 46]. In our study, one hedgehog gene (Unigene23139_All) was identified and highly expressed in umbonal and eyespots stages (Figure 9), suggesting that increased neural development was likely taking place during those stages.

The Wnt signal pathway has been shown to play an important role in the segmentation of the marine polychaete* Capitella capitata* [47, 48], while the maintenance of primitive hematopoiesis has been attributed to* Wnt4* in the vertebrate embryo [49]. Both* Wnt4* and* Wnt1* were observed in this study;* Wnt4* was highly expressed in the trochophore stage, while* Wnt1* was expressed in an increasing pattern over the course of larval development, suggesting possible involvement in blood formation from the beginning of development and in body transformation during all stages. Ten classes of fox genes were also found in our study, comprising the largest number of genes identified in the DEGs and displaying different trends in expression.* FoxL2*, XX-dominantly expressed in the differentiating ovaries of mammals [50], birds [51], and fish [52–54], was expressed highly in the D-shaped stage, suggesting the possible beginning of sexual development. Some important growth-related genes were also identified and differentially expressed among the five development stages, including* EGF-like*,* MAPK*, and* MAPKK *genes, which were actively expressed during the five developmental stages, and may contribute significantly to the transitions between developmental stages in* P. fucata* larvae.

Nonetheless, the body form transformations that take place during larval development involve a series of morphological and physiological changes and corresponding molecular changes, which have not been systematically studied and remain unclear. Therefore, a more broad understanding of the molecular underpinnings of important biological processes still merits further investigation.

## 5. Conclusions

In this study, a total of 174,126 unique transcripts were assembled and 60,999 were annotated. The number of unigenes varied between the five larval stages. The expression profiles of trochophore, D-shaped, and UES (umbonal, eyespots, and spats) larvae were distinctly different. Most unigenes were up- or downregulated in early stage transitions and 29 expression trends were sorted, eight of which were significant. In total, 80 development-related, differentially expressed unigenes were identified and eight were verified by qPCR. These observations should be helpful in understanding the molecular mechanisms of the larval metamorphic development of* P. fucata*.

## Supplementary Material

Supplement files Supplement table 1 GO function enrichment in significant trends Supplement table 2 KEGG pathway enrichment in significant trends

## Figures and Tables

**Table 1:** Primers for genes used for qPCR verification.

Trend	Unigene code	Annotated gene	Primer
0	Unigene27615_All	*Brachyury*	qBran-S: 5′ GCCAAAGAAAGACCAGAAGG 3′
			qBran-A: 5′ TCAGGCTAAGGCGATCACAA 3′
0	Unigene27517_All	*Six3*	qSix3-S: 5′ ATACAGGGTGAGGAAGAAGT 3′
			qSix3-A: 5′ TTATCTCCGCCTTGCTGTTG 3′
3	Unigene23318_All	*Engrailed*	qEng-S: 5′ TAGACAGAGCATCGCCTTTA 3′
			qEng-A: 5′ TTGTGATTTAACTGCCTGCT 3′
3	Unigene41009_All	*Pax-7*	qPax-S: 5′ GCGGAAACAGATGGGAAGCA 3′
			qPax-A: 5′ ACCGAATGACGGAAACGACT 3′
26	CL664.Crontig4_All	*MAPK*	qMAPK-S: 5′ TTTACTCCAAACAGCCCTAC 3′
			qMAPK-A: 5′ TTGCTATCTGGTCCACTTCA 3′
29	CL7953.Contig2_All	*Notch*	qNotch-S: 5′ CCAGCCACGGTATCCAAGTA 3′
			qNotch-A: 5′AGCCTCGAACAGAATATCCACT 3′
29	CL1306.Contig2_All	*Wnt 1*	qWnt1-S: 5′ TGATGCCTACGGTAAATACG 3′
			qWnt1-A: 5′ TAACCTTGAGGTGGGAGAAC 3′
29	Unigene27337_All	*Lox2*	qLox2-S: 5′ CTACCCGAGTTGAATGTGGG 3′
			qLox2-A: 5′ GAAAGTAAGACGGACGAGCC 3′

**Table 2:** Summary statistics of sequence assembly from 11 samples of the pearl oyster, *Pinctada fucata*.

Sample	Total raw reads	Total clean reads	Q20	Number of contigs	ContigN50	Number of unigenes	Mean length	N50
Gill	59,492,360	53,662,442	97.67%	215,808	380	113,516	592	977
Adductor muscle	58,602,062	53,889,264	97.42%	127,634	347	76,240	415	495
Hepatopancreas	57,854,582	52,672,774	97.75%	163,089	398	85,839	544	811
Pearl sac	56,926,624	52,155,950	97.49%	149,236	582	97,501	545	827
Mantle	58,610,258	52,433,102	97.72%	183,633	384	100,679	567	900
Hemocytes	54,707,500	51,751,784	98.54%	178,460	509	96,469	599	1025
Trochophore	59,887,806	54,413,910	97.96%	175,174	399	75,400	584	785
D-shaped	58,511,662	51,746,334	97.98%	190,135	485	88,830	626	886
Umbonal	66,858,916	55,046,652	97.88%	118,010	352	51,102	536	683
Eyespots	58,534,394	52,943,288	97.88%	182,671	504	84,045	649	937
Spats	60,561,378	54,999,258	97.85%	180,265	521	82,133	689	1022
All						174,126	866	1569

**Table 3:** Summarized statistics of the functional annotation in 11 samples of the pearl oyster, *Pinctada fucata*.

Database	Number of annotated genes
Nr annotation	38857
Swissprot annotation	49580
Annotation unigenes for KEGG	43753
Annotation unigenes for GO	13465
Annotation unigenes for COG	23754
CDS	60,946
CDS by ESTScan	13,966

Total	60999

**Table 4:** Top 26 KEGG pathways.

Pathway	Count (43,753)	Pathway ID
Metabolic pathways	5184	ko01100
Regulation of actin cytoskeleton	2302	ko04810
Vascular smooth muscle contraction	2176	ko04270
Focal adhesion	2142	ko04510
Pathways in cancer	1607	ko05200
Tight junction	1544	ko04530
Hypertrophic cardiomyopathy (HCM)	1426	ko05410
Dilated cardiomyopathy	1402	ko05414
Calcium signaling pathway	1401	ko04020
Amoebiasis	1384	ko05146
Tuberculosis	1379	ko05152
RNA transport	1336	ko03013
Salmonella infection	1333	ko05132
Neuroactive ligand-receptor interaction	1304	ko04080
Epstein-Barr virus infection	1240	ko05169
Phagosome	1239	ko04145
Spliceosome	1231	ko03040
Purine metabolism	1191	ko00230
*Vibrio cholera* infection	1157	ko05110
Endocytosis	1139	ko04144
Huntington's disease	1121	ko05016
Viral myocarditis	1090	ko05416
MAPK signaling pathway	1046	ko04010
Cardiac muscle contraction	1044	ko04260
Gastric acid secretion	1019	ko04971
Ubiquitin mediated proteolysis	1014	ko04120

**Table 5:** Early development-related DEGs and their expression trends in *Pinctada fucata *(80).

Unigene ID	Annotated gene	Reference species	*E* value	Trend
Unigene41154_All	Nanos-like protein 1	*Crassostrea gigas*	5.00*E* − 73	0
Unigene40485_All	EGF-like 4	*Crassostrea gigas*	9.00*E* − 41	0
Unigene27615_All	brachyury	*Saccostrea kegaki*	0	0
Unigene40515_All	TBX6	*Crassostrea gigas*	2.00*E* − 49	0
CL19363.Contig1_All	foxn1	*Homo sapiens*	2.00*E* − 48	0
Unigene36457_All	foxb1	*Crassostrea gigas*	6.00*E* − 129	0
Unigene47210_All	foxi1c	*Caenorhabditis brenneri*	4.00*E* − 16	0
Unigene49637_All	OAR	*Crassostrea gigas*	3*E* − 96	0
Unigene49559_All	notch-like protein 1	*Crassostrea gigas*	3.00*E* − 67	0
CL26075.Contig2_All	Cbx1	*Mus musculus*	5.00*E* − 60	0
CL4780.Contig2_All	XHOX-3	*Crassostrea gigas*	1*E* − 124	0
Unigene23054_All	ZF E-box-binding		4.00*E* − 60	0
Unigene30958_All	ZF protein 64		6.00*E* − 08	0
Unigene18460_All	aristaless-like 4		1*E* − 120	0
Unigene22708_All	ARX		4.00*E* − 87	0
Unigene22940_All	IRX-6		2*E* − 101	0
Unigene27129_All	engrailed		2.00*E* − 56	0
Unigene27119_All	homeobox-like protein		2.00*E* − 45	0
Unigene27517_All	SIX3		4*E* − 129	0
Unigene31497_All	ceh-9		4.00*E* − 64	0
Unigene31654_All	Slou		1*E* − 103	0
Unigene40727_All	unc-4-like protein		7*E* − 139	0
Unigene40815_All	homeobox 2		7*E* − 120	0
Unigene49664_All	even-skipped-like protein 1		2*E* − 107	0
Unigene50280_All	not2		9*E* − 88	0
Unigene23318_All	engrailed-2-B		8*E* − 61	3
Unigene51601_All	BarH-like 1		6*E* − 31	3
Unigene31090_All	PKNOX2		3*E* − 156	3
Unigene49991_All	HMX1		2*E* − 27	3
Unigene41009_All	Pax-7		4*E* − 128	3
Unigene17191_All	Pax-2-A		5*E* − 102	3
CL11027.Contig2_All	Polycomb BMI-1-A	*Danio rerio*	3.00*E* − 78	3
CL13897.Contig2_All	Polycomb suz12	*Xenopus tropicalis*	2.00*E* − 149	3
CL20556.Contig2_All	pcgf3	*Xenopus tropicalis*	1.00*E* − 77	3
CL26777.Contig2_All	EPC1	*Homo sapiens*	4.00*E* − 147	3
CL15780.Contig2_All	Wnt-4	*Homo sapiens*	3.00*E* − 93	3
Unigene44847_All	BMP 3/3b	*Branchiostoma japonicus*	2.00*E* − 59	3
CL11806.Contig6_All	FOXP1	*Homo sapiens*	5.00*E* − 104	3
Unigene32767_All	foxe1	*Crassostrea gigas*	3.00*E* − 81	3
CL4477.Contig6_All	Msx2	*Crassostrea gigas*	1.00*E* − 22	3
Unigene45481_All	ceh-37		2*E* − 59	12
Unigene55326_All	Pax-8		7*E* − 59	20
Unigene18153_All	corepressor 1-like		0	21
Unigene45344_All	SIX4		7*E* − 84	21
Unigene45422_All	aristaless		5*E* − 72	21
Unigene45788_All	HMX3-B		1*E* − 55	21
Unigene40909_All	odd-skipped-related 1	*Crassostrea gigas*	7.00*E* − 74	21
Unigene22645_All	Hox5	*Haliotis rufescens*	3.00*E* − 85	24
Unigene35405_All	EGF-like 1	*Crassostrea gigas*	6.00*E* − 66	24
Unigene17944_All	foxc2	*Crassostrea gigas*	8.00*E* − 174	24
Unigene45321_All	Dorsal root ganglia		2*E* − 123	26
CL664.Contig4_All	MAPK	*Homo sapiens*	7.00*E* − 127	26
Unigene23113_All	LIM		7*E* − 122	27
Unigene44988_All	DBX1-A		3*E* − 93	27
CL19911.Contig2_All	foxg1	*Xenopus laevis*	5.00*E* − 25	27
Unigene17797_All	Nkx-2.2a		2*E* − 137	28
Unigene44641_All	Nkx-6.2		1*E* − 125	28
Unigene22323_All	hhex		3*E* − 92	28
Unigene22601_All	Xlox	*Euprymna scolopes*	5.00*E* − 55	28
Unigene33207_All	GBX-1		3.00*E* − 07	28
Unigene36590_All	HOX3		8*E* − 89	28
Unigene45891_All	MOX-2		9*E* − 82	28
CL20549.Contig2_All	bicaudal	*Xenopus laevis*	1.00*E* − 45	28
Unigene23139_All	hedgehog	*Crassostrea gigas*	5.00*E* − 99	28
CL13232.Contig2_All	EGF-like D1044.2	*Caenorhabditis elegans*	9.00*E* − 06	28
CL178.Contig1_All	EGFR	*Apis mellifera*	0	28
CL5633.Contig3_All	MAPKAPK5	*Homo sapiens*	5.00*E* − 132	28
Unigene22098_All	O-fut1	*Crassostrea gigas*	1.00*E* − 131	28
Unigene31699_All	foxl2	*Crassostrea gigas*	1.00*E* − 119	28
Unigene36180_All	foxl1	*Crassostrea gigas*	1.00*E* − 119	28
Unigene40504_All	foxslp2	*Crassostrea gigas*	8.00*E* − 116	28
Unigene31565_All	bmp 2b	*Crassostrea gigas*	6.00*E* − 96	29
CL7953.Contig2_All	Notch	*Crassostrea gigas*	1.00*E* − 139	29
Unigene36286_All	Notch-like protein 2	*Crassostrea gigas*	5.00*E* − 65	29
Unigene27672_All	ZF C2H2	*Brugia malayi*	4.00*E* − 07	29
Unigene27337_All	LOX2		8.00*E* − 98	29
Unigene27686_All	BarH-like 1-like	*Oreochromis niloticus*	3.00*E* − 36	29
CL12322.Contig2_All	ALDH16A1	*Bos taurus*	1.00*E* − 179	29
CL3565.Contig3_All	ALDH2	*Crassostrea gigas*	0	29
CL1306.Contig2_All	Wnt 1	*Homo sapiens*	7.00*E* − 13	29

## Additional Points

*Highlights. *(i) A large number of assembled transcripts from* Pinctada fucata *are reported for the first time. (ii) Large variations in expression of DEGs related to development were observed in early larval stages. (iii) Twenty-nine expression trends were identified for the first time.
